# SHR/NCrl rats as a model of ADHD can be discriminated from controls based on their brain, blood, or urine metabolomes

**DOI:** 10.1038/s41398-021-01344-4

**Published:** 2021-04-22

**Authors:** Camille Dupuy, Pierre Castelnau, Sylvie Mavel, Antoine Lefevre, Lydie Nadal-Desbarats, Sylvie Bodard, Julie Busson, Diane Dufour-Rainfray, Helene Blasco, Patrick Emond, Laurent Galineau

**Affiliations:** 1 UMR 1253, iBrain, Université de Tours, Inserm, Tours, France; 2 CHRU Tours, Tours, France

**Keywords:** Diagnostic markers, Molecular neuroscience, ADHD

## Abstract

Attention-Deficit Hyperactivity Disorder (ADHD) is one of the most common neurodevelopmental disorder characterized by inattention, impulsivity, and hyperactivity. The neurobiological mechanisms underlying ADHD are still poorly understood, and its diagnosis remains difficult due to its heterogeneity. Metabolomics is a recent strategy for the holistic exploration of metabolism and is well suited for investigating the pathophysiology of diseases and finding molecular biomarkers. A few clinical metabolomic studies have been performed on peripheral samples from ADHD patients but are limited by their access to the brain. Here, we investigated the brain, blood, and urine metabolomes of SHR/NCrl vs WKY/NHsd rats to better understand the neurobiology and to find potential peripheral biomarkers underlying the ADHD-like phenotype of this animal model. We showed that SHR/NCrl rats can be differentiated from controls based on their brain, blood, and urine metabolomes. In the brain, SHR/NCrl rats displayed modifications in metabolic pathways related to energy metabolism and oxidative stress further supporting their importance in the pathophysiology of ADHD bringing news arguments in favor of the Neuroenergetic theory of ADHD. Besides, the peripheral metabolome of SHR/NCrl rats also shared more than half of these differences further supporting the importance of looking at multiple matrices to characterize a pathophysiological condition of an individual. This also stresses out the importance of investigating the peripheral energy and oxidative stress metabolic pathways in the search of biomarkers of ADHD.

## Introduction

Attention-deficit/hyperactivity disorder (ADHD) is one of the most common neurodevelopmental disorder affecting 5.3% of school-aged children^1^ and 2.5–4.9% of adults^2,3^. ADHD symptoms include inattention and hyperactivity/impulsivity, with three symptom presentations described by the Diagnostic and Statistical Manual of Mental Disorders, Fifth Edition (DSM-5): predominantly inattentive, predominantly hyperactive/impulsive, and combined profiles, which may vary over time. The diagnosis mainly relies on detailed clinical evaluations of the symptoms and careful assessment of the attention and executive functions^4^. However, the high heterogeneities of the phenotypic presentations and temporal courses of the disorder, along with the numerous comorbidities, render this diagnosis difficult to establish and often delayed^5^. Besides, false positives and false negatives are not unusual. Thus, there is a crucial need for biomarkers to facilitate an early and reproducible ADHD diagnosis in order to promote appropriate care and follow-up of the patients. Toward this aim, numerous genetic and neuroimaging studies were conducted in ADHD patients over the past two decades but failed to provide relevant tools for reliable diagnosis on a routine basis^4^.

To date, metabolomic strategies remain largely unexplored in the search for ADHD biomarkers^6^. Only a few hypothesis-driven and one nonhypothesis-driven studies using various sample types have been performed in ADHD patients. Therefore, additional studies are needed to identify useful peripheral biomarkers^7,8^. Metabolomics aims at providing qualitative and quantitative changes of hundreds of metabolites^9^ and has been increasingly used to identify molecular fingerprints in neurodevelopmental disorders. In this context, metabolomic studies in animal models are of a great interest as it allows to access peripheral but also central compartments to better evaluate how potential peripheral metabolomic changes can reflect central modifications.

The goal of the present study was to explore the cerebral and peripheral metabolomes of SHR/NCrl vs WKY/NHsd rats, which is one of the most extensively studied and characterized animal model of ADHD with good face and construct validities^10–15^. Our specific objectives were (i) to characterize the modifications in the brain, blood, and urine metabolomes in this animal model of ADHD, and (ii) to identify peripheral modifications in metabolites/metabolic pathways related to the reported cerebral changes.

## Materials and methods

### Subjects

The subjects were eight male SHR/NCrl rats (Charles River, Germany), which were compared to eight male WKY/NHsd control rats (Harlan, United Kingdom); all rats were 8 weeks old, i.e., before the onset of hypertension in the SHR/NCrl rats, as described by Sagvolden et al.^11^. Animals were housed in groups of 2 or 3 under humidity—and temperature-controlled conditions and a 12:12 light-dark cycle (light on at 7:00 AM) with ad libitum access to food and water. All animal experiments were conducted in accordance with EU guidelines (EU Directive 2010/63/EU for animal experiments), and approved by a local Ethics Committee (Comité d’Ethique en Expérimentation Animale—Pays de la Loire—France).

### Sample collection

Urine samples were collected before sacrifice by handling the animals. Immediately after decapitation, blood samples were collected in vials filled with 1.8 mg of EDTA (Sigma–Aldrich, France), centrifuged at 11,500 × *g* and 4 °C for 10 min and the plasma retrieved. Samples were stored at −80 °C. The brains were quickly removed and prepared as described below.

### Sample preparation

Brain samples were freeze-dried (FreeZone® 4.5 L, Labconco, USA) at −107 °C and 0.2 mbar for 12 h. The lyophilized material (3 mg) was mixed with 750 µL of ACN/H_2_O (1:1), vortexed for 15 s, homogenized by gentle planar shaking at 4 °C for 30 min and centrifuged at 11,500 × *g* and 4 °C for 15 min (Thermo Fischer, USA). Supernatants (500 µL) were concentrated under vacuum (SpeedVac, Thermo Fischer, USA) at 45 °C for 4 h. The resultant dry residues were reconstituted in 100 µL of MeOH/H_2_O (1:9).

Urine samples were diluted to the tenth with ultrapure water and vortexed for 15 s.

Metabolites were extracted from 100 µL of plasma by adding 800 µL of MeOH/H_2_O (1:1). The samples were then vortexed for 15 s, homogenized by gentle planar shaking at 4 °C for 30 min, and centrifugated at 11,500 × *g* and 4 °C for 10 min to collect 500 µL of supernatant. Supernatants were concentrated under vacuum (SpeedVac, Thermo Fischer, USA) at 45 °C for 4 h. The dried residues were reconstituted in 100 µL of MeOH/H_2_O (1:9). Each prepared sample was then transferred to a 96-well plate for liquid chromatography-high resolution mass spectrometry (LC-HRMS) analysis. Quality control samples (QCs) were obtained from a pooled mixture of equal volumes of all samples for each matrix. Fifteen QCs were injected to equilibrate the chromatographic system before each analytic batch and QCs were analyzed every 10 samples.

### Liquid Chromatography-High Resolution Mass Spectrometry Analysis (LC-HRMS)

LC-HRMS analysis was performed on a UPLC Ultimate 3000 system (Dionex, Germany) coupled to a Q-Exactive mass spectrometer (Thermo Fisher, Germany) and operated in positive and negative electro spray ionization (ESI+ and ESI−). Chromatography was performed as previously described^16^. For both modes, heated ESI source parameters consisted in a spray voltage of 3 kV, a capillary temperature of 380 °C, a heater temperature of 350 °C, a sheath gas flow of 40 arbitrary units (AU), an auxiliary gas flow of 20 AU, a spare gas flow of 2 AU, and a tube lens voltage of 50 V. The full-scan acquisition ranged from 58 to 870 *m/z* and the instrument operated at 70,000 resolution (*m/z* = 200), with an automatic gain control target of 1 × 10^6^ charges and a maximum injection time of 250 ms. The identity of each sample was processed by experimenters blinded its experimental group.

### Data processing for targeted LC-HRMS

A library of 495 standard metabolites (Mass Spectroscopy Metabolite Library, IROA technologies, USA) was used for the construction of an in-house metabolite database. Targeted molecules from our database were selected and integrated into Xcalibur 2.2 (Thermo Fisher, USA) as previously described^17^. Each peak area was normalized to the total of peak areas of interest. Metabolites detected with greater than 30% variance (CV%) in QC samples were not selected for further analysis.

### Statistical analysis

The metabolic card was performed using the web-based tool iPath 3.0 to represent all the metabolites detected in the brain on the metabolic pathways based on the latest version of the Kyoto Encyclopedia of Genes and Genomes (KEGG, www.kegg.jp). A Venn diagram was performed to assess the complementarity between the rat’s brain, plasma, and urines samples using the jvenn online tool (http://jvenn.toulouse.inra.fr/app/index.html).

Multivariate analyses were performed using Metaboanalyst 4.0^18^, and included principal component analyses (PCA) and partial least squares discriminant analyses (PLS-DA). PCA were performed on brain, plasma, and urine samples to identify outliers (one rat from each group of the brain samples was excluded), and to perform unsupervised clustering of SHR/NCrl and WKY/NHsd rats. PLS-DA were performed on brain, plasma, and urine samples to build discriminant models between the two strains. Each model was characterized by the cumulative modeled variation in the Y matrix *R*^2^Y, and the cross-validated predictive ability *Q*^2^ values, providing the discriminant metabolites called variable importance in projection (VIP). Metaboanalyst 4.0 was also used for univariate analyses using volcano plots on brain, plasma, and urine samples to select the metabolites whose levels were statistically significantly different between SHR/NCrl and WKY/NHsd rats (*p*-value corrected for False Discovery Rate = [FDR] values), and whose concentrations differed between SHR/NCrl rats and controls by less than 0.75-fold or more than 1.25-fold. Pathway analyses using Metaboanalyst 4.0 were conducted on the VIP metabolites. The pathways were considered when displaying FDR values <0.05 and a non-null impact value. Correlation analyses were performed for metabolites that were significantly altered in the brain and plasma and/or urines samples using Metaboanalyst 4.0 and were considered significant when *p* < 0.05. Linear regression curves were generated using GraphPad Prism 7 (GraphPad Prism, USA).

## Results

### Metabolic profile of the central and peripheral compartments of SHR/NCrl and WKY/NHsd rats

**Fig. 1:**
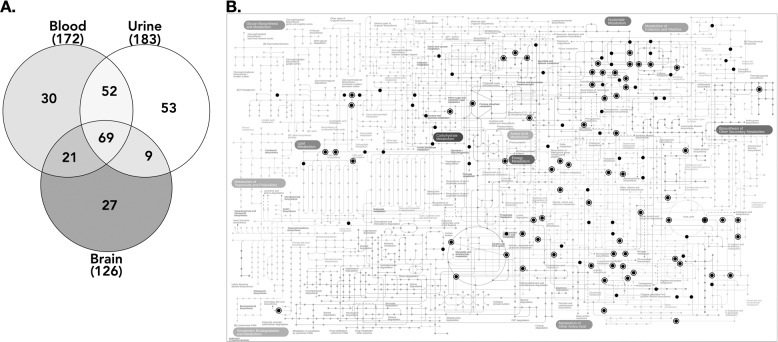
Number of metabolites detected in the brain, blood, and urines of SHR/NCrl and WKY/NHsd rats. **A** Venn diagram presenting the number of metabolites detected in each compartment. **B** Black circles representing metabolites detected in the blood or urine compartments of SHR/NCrl and WKY/NHsd rats. The open circles correspond to the metabolites detected in the brain of SHR/NCrl and WKY/NHsd rats.

Starting from a library of 495 compounds, the number of metabolites detected in both rat strains ranged from 125 to 182, representing 25–37% of the whole library (Fig. 1A). The number of metabolites detected in the brain was slightly smaller compared to the peripheral compartments (125 metabolites in the brain vs 171 and 182 in blood and urine, respectively; Fig. 1A). In addition, the brain shared 77 metabolites (61%) with the urine compartment, and 89 (71%) with the blood compartment (Fig. 1A). Finally, 68 metabolites (55% of the cerebral metabolites) were detected in all the matrices (Fig. 1A). Based on pathway analyses performed on each dataset, the detected metabolites were involved in 47, 50, and 51 metabolic pathways, respectively, described in the rat urine, blood, and brain compartments (Fig. 1B).

### SHR/NCrl rats can be differentiated from controls based on their cerebral metabolome

**Fig. 2:**
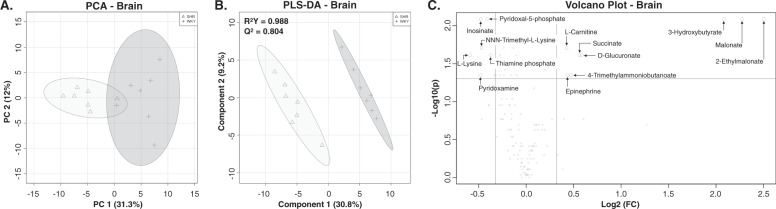
Distinction between the SHR/NCrl and WKY/NHsd rats (*n* = 7 per group) based on brain metabolome. **A** Principal component analysis (PCA) score plot differentiating two groups corresponding to the brain metabolomes of SHR/NCrl (triangles) and WKY/NHsd (crosses) rats. **B** Partial least squares discriminant analysis (PLS-DA) showing the discriminant model based on two components built to separate SHR/NCrl (triangles) from WKY/NHsd brain metabolomes (crosses). **C** Volcano plot showing the metabolites with significant FDR values between SHR/NCrl and WKY/NHsd rats and fold differences lower than 0.75 or greater than 1.25.

Applying a PCA to the 125 metabolites detected in the brain enabled to clearly differentiate the SHR/NCrl rats from controls using two components representing 43% of their overall variations between the two strains (Fig. 2A). In addition, the distance between the subjects in the SHR/NCrl group suggests a low interindividual variability of their brain metabolome (Fig. 2A). The SHR/NCrl rats were separated from controls using a PLS-DA analysis based on a model showing an accuracy of 0.929, a R^2^Y value of 0.988, and a predictive ability (Q^2^) of 0.804 for the two first components (Fig. 2B). Fifty-three metabolites showed variable importance in prediction (VIP) scores superior to 1 (Table S1). Among them, 31 displayed significantly different brain concentrations in SHR/NCrl rats vs controls (Table S1). Of these 31 metabolites, 14 had concentrations that differed between SHR/NCrl rats and controls by less than 0.75-fold or more than 1.25-fold (Fig. 2C, Table S1). In detail, the brain concentrations of 2-ethylmalonate, malonate, 3-hydroxybutyrate, glucuronate, succinate, 4-trimethylammoniobutanoate, epinephrine, and carnitine were significantly higher in SHR/NCrl rats vs controls, while the concentrations of lysine, pyridoxamine, inosinate, NNN-trimethyl-lysine, pyridoxal-5-phosphate, and thiamine phosphate were significantly lower (Table S1). A pathway analysis performed using the 31 significantly different metabolites revealed modifications in 14 metabolic pathways between SHR/NCrl and control animals (Table 1). Among them, five pathways were related to amino acids metabolism (alanine and beta-alanine metabolism; aspartate and glutamate metabolism; lysine degradation; arginine and proline metabolism and tyrosine metabolism), four to energy metabolism (pantothenate and CoA biosynthesis; butanoate metabolism; citrate cycle and pentose and glucuronate interconversions), three to nucleotide metabolism (purine, pyrimidine and nicotinate metabolisms), and two to vitamin metabolism (vitamin B6 and ascorbate metabolisms) (Table 1). This pathway analysis did not include five metabolites of the VIPs, which are also involved in energy (2-ethylmalonate, malonate, 3-hydroxybutyrate, and carnitine), and vitamin (thiamine phosphate) metabolisms.

**Table 1:** Significant brain, blood, and urine pathways involved in the distinction between SHR/NCrl and WKY/NHsd rats based on the variable importance in projection (discriminant metabolites) determined by the PLS-DA.

Compartment	Significant metabolic pathways		Match status	Matching metabolites	Holm p	FDR	Impact
Brain	Amino acid metabolism	Beta-Alanine metabolism	1/21	3-Ureidopropionate, Aspartate	0.0006	0.0001	0.104
		Alanine, aspartate, and glutamate metabolism	5/28	N-Acetyl-Aspartate, Aspartte, Asparagine, 4-Aminobutanoate, **Succinate**	0.0001	0.0001	0.400
		Lysine degradation	3/25	**Lysine**, **4-Trimethylammoniobutanoate**, **NNN-Trimethyl-Lysine**	0.0026	0.0005	0.005
		Arginine and proline metabolism	2/38	4-Aminobutanoate, 4-Guanidinobutanoate	0.0011	0.0003	0.024
		Tyrosine metabolism	1/42	**Epinephrine**	0.0089	0.0019	0.012
	Energy metabolism	Pantothenate and CoA biosynthesis	1/19	3-Ureidopropionate, Aspartate	0.0006	0.0001	0.029
		Butanoate metabolism	3/15	Hydroxybutanoate, 4-Aminobutanoate, **Succinate**	0.0005	0.0001	0.032
		Citrate cycle (TCA cycle)	1/20	**Succinate**	0.0059	0.0009	0.033
		Pentose and glucuronate interconversions	1/18	**Glucuronate**	0.0067	0.0010	0.125
	Nucleotide metabolism	Pyrimidine metabolism	1/39	3-Ureidopropionate	<0.0001	<0.0001	0.013
		Purine metabolism	2/66	**Inosinate**, Adenine	0.0010	0.0025	0.130
		Nicotinate and nicotinamide metabolism	3/15	Aspartate, Nicotinamide, Nicotinate	0.1231	0.0476	0.194
	Vitamin metabolism	Vitamin B6 metabolism	2/9	**Pyridoxamine, Pyridoxal-5-phosphate**	0.0018	0.0003	0.333
		Ascorbate and aldarate metabolism	1/10	**Glucuronate**	0.0068	0.0010	0.250
Blood	Amino acid metabolism	Beta-Alanine metabolism	4/21	Beta-Alanine, Beta-alanyl-N-methyl-histidine, Dihydrouracil, Spermidine	0.0014	0.0002	0.455
		Alanine, aspartate and glutamate metabolism	5/28	Alanine, Asparagine, 4-Aminobutanoate, Glutamine, Succinate	<0.0001	<0.0001	0.200
		Phenylalanine, tyrosine and tryptophan biosynthesis	1/4	Phenylalanine	0.0170	0.0019	0.500
		Arginine and proline metabolism	3/38	4-Aminobutanoate, Creatine, Spermidine	<0.0001	<0.0001	0.070
		Tryptophan metabolism	5/41	Indole-3-Acetaldehyde, Kynurenine, N-Acetylserotonin, N-Methyltryptamine, Tryptophan	<0.0001	<0.0001	0.291
		Glycine, serine, threonine metabolism	2/34	Creatine, Threonine	0.0036	0.0005	0.024
		Histidine metabolism	2/16	Beta-Alanyl-N-Methyl-Histidine, N-Methyl-Histidine	<0.0001	<0.0001	0.049
	Energy metabolism	Pantothenate and coA biosynthesis	3/19	Panthotenate, Dihydrouracil, Beta-Alanine	0.2713	0.0426	0.500
		Butanoate metabolism	2/15	4-Aminobutanoate, Succinate	0.0237	0.0032	0.032
		Pentose and glucuronate interconversions	1/18	Glucuronate	0.0052	0.0010	0.125
	Nucleotide metabolism	Pyrimidine metabolism	4/39	Beta-Alanine, Dihydrouracil, Glutamine, Thymine	0.0002	<0.0001	0.063
		Caffeine metabolism	1/12	Dimethylxanthine	0.0011	0.0015	0.692
	Vitamin metabolism	Vitamin B6 metabolism	1/9	Pyridoxamine	0.2113	0.0426	0.078
	Lipid metabolism	Sphingolipid metabolism	1/21	Sphinganine	0.1564	0.0193	0.154
Urine	Amino acid metabolism	Beta-alanine metabolism	2/21	3-Ureidopropionate, Aspartate	0.0028	0.0014	0.105
		Alanine, aspartate and glutamate metabolism	3/28	Asparagine, Aspartate, Glutamate	0.0011	0.0001	0.420
		Cysteine and methionine metabolism	1/33	Serine	0.0018	0.0004	0.022
		Arginine and proline metabolism	5/38	Arginine, Glutamate, Guanidinoacetate, Hydroxyproline, Proline	<0.0001	<0.0001	0.306
		Glutamate and glutamine metabolism	1/6	Glutamate	0.0016	0.0002	0.500
		Glycine, serine, threonine metabolism	3/34	5-Aminolevulinate, Guanidinoacetate, Serine	<0.0001	<0.0001	0.231
		Histidine metabolism	4/16	Aspartate, Glutamate, Histamine, N-Methyl-Histidiine	<0.0001	<0.0001	0.189
		Arginine biosynthesis	4/14	Arginine, Aspartate, Glutamate, N-Acetyl-Glutamate	0.0015	0.0002	0.193
	Energy metabolism	Pantothenate and coA biosynthesis	2/19	3-Ureidopropionate, Aspartate	0.0028	0.0014	0.029
		Glyoxylate metabolism	2/32	Glutamate, Serine	0.0005	<0.0001	0.042
		Sucrose metabolism	1/18	Glucose	0.0011	0.0001	0.421
		Pentose and glucuronate interconversions	1/18	Glucuronate	0.0016	0.0002	0.125
		Galactose metabolism	1/27	Glucose	0.0011	0.0001	0.035
	Nucleotide metabolism	Pyrimidine metabolism	1/39	3-Ureidoproppionate	0.0016	0.0002	0.013
		Caffeine metabolism	1/12	Dimethylxanthine	0.0011	0.0015	0.693
	Vitamin metabolism	Ascorbate metabolism	1/10	Glucuronate	0.0016	0.0002	0.250
	Antioxidative metabolism	Glutathione metabolism	1/28	Glutamate	0.0016	0.0002	0.020
		Porphyrin metabolism	2/30	5-Aminolevulinate, Glutamate	0.0003	<0.0001	0.028

For the brain, the metabolites written in bold characters correspond to those showing significant FDR and fold difference values.

### SHR/NCrl rats can also be differentiated from controls based on their peripheral metabolomes

**Fig. 3:**
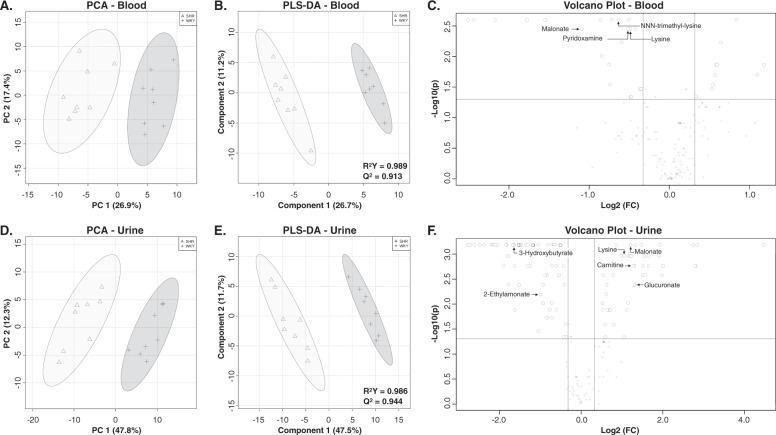
Distinction between SHR/NCrl and WKY/NHsd rats (*n* = 8 per group) based on peripheral metabolomes. **A** PCA score plot differentiating two groups corresponding to the blood metabolomes of SHR/NCrl (triangles) and WKY/NHsd (crosses) rats. **B** PLS-DA showing the discriminant model based on two components built to separate SHR/NCrl (triangles) from WKY/NHsd blood metabolomes (crosses). **C** Volcano plot showing the significant metabolites in the brain that also present significant FDR values between SHR/NCrl and WKY/NHsd rats and fold differences lower than 0.75 or greater than 1.25 in blood samples. **D** PCA score plot differentiating two groups corresponding to the urine metabolomes of SHR/NCrl (triangles) and WKY/NHsd (crosses) rats. **E** PLS-DA showing the discriminant model based on two components built to separate SHR/NCrl (triangles) from WKY/NHsd urine metabolomes (crosses). **F** Volcano plot showing the significant metabolites in the brain that also present significant FDR values between SHR/NCrl and WKY/NHsd rats and fold differences lower than 0.75 or greater than 1.25 in urine samples.

Applying a PCA to the metabolites detected in the blood, and urine enabled to clearly differentiate the SHR/NCrl rats from the controls (Fig. 3A, D). Based on the metabolites detected in the blood, the PCA analysis differentiated the SHR/NCrl rats *vs* controls using two components representing 44% of the overall variation between the two strains (Fig. 3A). Similarly, the PCA based on the metabolites detected in the urine compartment enabled to easily divide the animals into two distinct populations corresponding to SHR/NCrl and control rats (Fig. 3D). This was achieved using two components representing 60% of the overall variation existing between the two strains (Fig. 3D).

The SHR/NCrl rats were also differentiated from controls using a PLS-DA based on blood and urine metabolites (Fig. 3B, E). The model obtained on the blood metabolites had an accuracy of 1.0, a R^2^Y value of 0.989, and a Q^2^ of 0.913 for two components (Fig. 3B). The model obtained on urine metabolites showed an accuracy of 1.0, a R^2^Y value of 0.986, and a Q^2^ of 0.944 for two components (Fig. 3E). Sixty-seven VIPs were observed for the blood compartment including 41 metabolites with statistically significant distinct concentrations between SHR/NCrl rats and controls (Table S2). A total of 105 metabolites (including 90 VIPs) showed significantly different urine concentrations between SHR/NCrl rats and controls (Table S3).

**Fig. 4:**
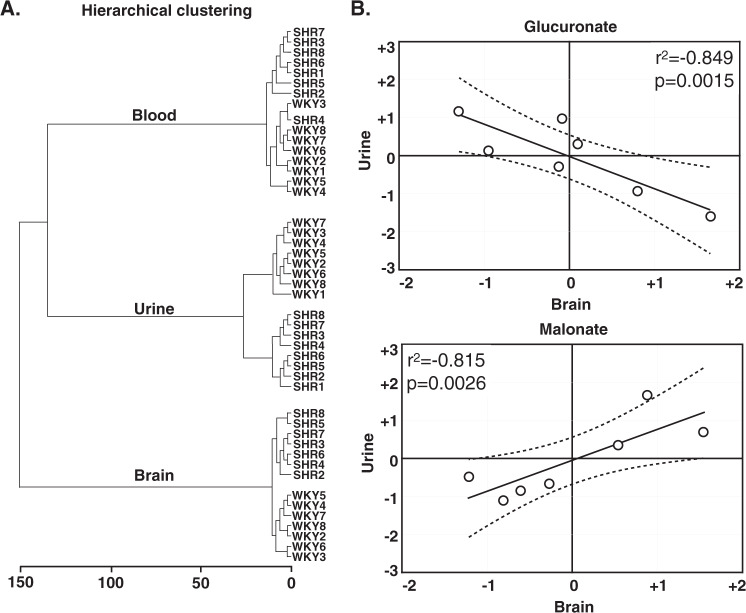
Relationships between the brain and peripheral metabolomes. **A** Hierarchical clustering analysis showing differences between brain and peripheral metabolomes in SHR/NCrl and WKY/NHsd rats. **B** Correlation between brain and peripheral concentrations of glucuronate and malonate in SHR/NCrl rats.

The pathway analysis showed significant modifications in 14 and 18 metabolic pathways in blood and urines of SHR/NCrl rats, respectively (Table 1). Interestingly, more than half of the pathways detected in the brain were also observed in the periphery (i.e., significant changes in 8 of the 14 brain pathways were found in the periphery; Fig. 4B). Thus, SHR/NCrl rats showed significant modifications in the amino acids, energy, nucleotides, and vitamin metabolisms in all the peripheral compartments as observed in the brain, as well as changes in the lipid metabolism in the blood and antioxidant metabolism in urines (Table 1).

Only looking at metabolites with significant FDR and fold-change values between SHR/NCrl and WKY/NHsd rats, six metabolites were significantly modified in both brain and urine compartments, and four were significantly modified in both brain and blood compartments. Malonate and lysine concentrations between SHR/NCrl and WKY/NHsd rats were different in brain, blood, and urines. Changes in pyridoxamine and NNN-trimethyl-lysine between SHR/NCrl vs WKY/NHsd rats were specific to the brain and blood compartments, while changes in 2-ethylmalonate, 3-hydroxybutyrate, carnitine, and glucuronate were specific to brain and urines compartments.

### Relationships between the brain and peripheral metabolomes

A hierarchical clustering revealed that the compartment has more impact than the strain on the metabolomic differences observed, with a clear distinction of the central vs peripheral metabolomes (Fig. 4A). Significant correlations between brain and peripheral concentrations of gluruconate and malonate were specifically observed in SHR/NCrl rats (Fig. 4B). A significant negative correlation between the levels of glucuronate in the brain and in the urines (r^2^ = 0.849, *p* = 0.002) (Fig. 4B). In addition, malonate concentrations in brain and urines were positively correlated to each other in SHR/NCrl rats (r^2^ = 0.815, *p* = 0.026) (Fig. 4B).

## Discussion

This study provides the first metabolomic characterization of the ADHD model based on the comparison of SHR/NCrl rats vs WKY/NHsd animals used as controls. Here we extensively studied the differences existing between SHR/NCrl rats vs controls in the brain and in the peripheral matrices. We showed that the brain, blood, and urine metabolomes can be used to discriminate SHR/NCrl rats from controls. The main cerebral differences between the two strains mainly consist in modifications in amino acid, energy, and vitamin metabolisms, that were also observed in blood and urines. In addition, malonate and glucuronate concentrations in the brain and urine compartments of SHR/NCrl rats were significantly correlated.

### Limitations of the animal model

Even though SHR compared to WKY rats are one of the mostly used animal model of ADHD, it has also been criticized for its face and predictive validities, based on studies showing no differences in locomotor activity between SHR rats and controls, and others suggesting a lack of effect of methylphenidate on the ADHD-related phenotypes of this animal model^19–23^. Sagvolden and collaborators reported genetic and behavioral differences in existing among the strains of SHR and WKY rats, and showed the importance of using specifically SHR rats from Charles River Germany (SHR/NCrl) compared to WKY rats from Envigo U.K. (WKY/NHsd) to model the ADHD-related inattention, impulsivity, and hyperactivity^11^. As also highlighted by Sagvolden and collaborators, most of the studies questioning the face and predictive validities of SHR/NCrl vs WKY/NHsd rats as a model of ADHD were confounded by the settings used to assess rat’s behaviors^11^. In this study, we used the recommended strains of SHR and WKY rats to match with the studies that extensively characterized the ADHD-related behaviors of the animal model.

This animal model of ADHD is also confounded by the development of hypertension from 10 to 12 weeks of age^24^, and cognitive deficits observed in 11-week-old animals^25^. A recent metabolomic study performed in urine and plasma samples of 40-weeks-old SHR and WKY rats described a significant correlation between urine and blood variations of several metabolites and hypertension^26^ (urine levels of dimethylarginine, N2-acetyl-L-ornithine, buthionine sulfoximine, uric acid, L-isoleucine, and creatinine; blood levels of Vitamine E and phospholipids). Although we cannot completely rule out that our metabolic data could be related to the development of hypertension in our experimental conditions, we worked on 8-weeks-old animals, and our peripheral data are not in line with the urinary and plasma biomarkers significantly correlated with hypertension in these studies.

### SHR/NCrl rats mainly differed from controls through their cerebral energy and adrenaline metabolisms

SHR/NCrl rats showed lower brain levels in thiamine phosphate compared to controls. Interestingly, old reports suggested that vitamin supplementations, including thiamine, could carry beneficial effects on ADHD symptoms^27,28^. Thiamine phosphate is a cofactor of the pyruvate dehydrogenase, alpha-ketoglutarate dehydrogenase, and trans-ketolase^29^, involved in the glycolysis, pentose phosphate pathway and Krebs cycle. Therefore, significant alterations of the cerebral energy metabolism were expected in this ADHD animal model. This is consistent with studies reporting decreased expression of the dihydrolipoyl dehydrogenase, a part of the pyruvate dehydrogenase complex in the medulla oblongata of SHR/NCrl rats^30^, along with decreased ATP production capacities in discrete brain regions in this animal model of ADHD^30,31^.

In line with such hypothesis, we observed significantly elevated levels in succinate, malonate and 2-ethylmalonate in SHR/NCrl rats compared to controls. Both malonate and 2-ethylmalonate inhibit several Krebs cycle’s enzymes, including the succinate dehydrogenase^32,33^ fitting well with the higher levels of succinate detected. Based on the key role of succinate in the Krebs cycle and in the respiratory chain, these results further support a dysfunctional brain energy metabolism in this animal model of ADHD. In addition, elevated succinate levels and mitochondrial dysfunctions have been associated to an increased oxidative stress, which has been extensively described in the brain of this animal model^34,35^, and in ADHD patients^36–39^. The higher brain levels of 2-ethylmalonate also observed in SHR/NCrl rats are consistent with an increased oxidative stress as it is a reliable lipid peroxidation biomarker^40^. Thus, the higher brain levels of glucuronate detected could reflect an elevated activity of the pentose phosphate pathway due to the increased oxidative stress, as the pentose phosphate pathway is used by neurons to regenerate glutathione^41^. In line with these results and other studies reporting protective effects of methylphenidate on oxidative stress^37,38^, Fagundes and collaborators already showed that a methylphenidate treatment increases the succinate dehydrogenase and activates the mitochondrial respiratory chain of young Wistar rats further supporting our hypothesis^42^.

We also detected higher carnitine and 3-hydroxybutyrate brain levels in SHR/NCrl rats *vs* controls. Carnitine plays a key role in energy metabolism by transporting long-chain fatty acids to the mitochondria for the production of acetyl-CoA via the beta-oxidation, and removes short and medium chain fatty acids formed by metabolic processes to prevent their accumulation in the mitochondria^43^. Thus, these higher brain carnitine levels could reflect modifications in the lipid metabolism in SHR/NCrl rats. Fatty acids and ketone bodies such as 3-hydroxybutyrate are alternative energy substrates during development or when glucose availability is limited^31^. In our experimental conditions, the cerebral glucose concentrations in the SHR/NCrl rats were not different from controls. Moreover, 3-hydroxybutyrate is no longer a substitute of glucose for brain energy metabolism after 30 days of life in rats^44^. Studies reported a switch towards other energy substrates to maintain the Krebs cycle supply with acetyl-CoA in conditions of decreased pyruvate dehydrogenase activity^45–47^. Based on the lower thiamine levels we observed in SHR/NCrl rats, and on its role as cofactor of enzymes including the pyruvate dehydrogenase, we cannot exclude that the higher carnitine and 3-hydroxybutyrate levels we observed could reflect such a switch in energy substrates. Another explanation could rely in their antioxidant properties^43,48–50^ in response to the higher oxidative stress detected in this animal model of ADHD^34,35^.

Intracerebral injections of malonate have been used as an inducer of energy mitochondrial metabolic dysfunctions, and have been shown to induce modifications in dopamine neurotransmission^51^. Based on the implication of dopamine in the pathophysiology of ADHD, this support that such abnormalities related to neuroenergetic^52–54^ and oxidative stress could be one of the molecular mechanism involved in the pathophysiology of ADHD-related phenotypes in SHR/NCrl vs WKY/NHsd rats. Thus, we expected to see modifications in the cerebral levels of various neurotransmitters in SHR/NCrl rats vs controls, as the implication of multiple neurotransmitters has already been suggested in ADHD^55–57^, and dopamine along with noradrenaline modifications have already been described in discrete regions in this animal model^13^. The fact that we analyzed the whole brain rather than discrete regions could explain the lack of results related to neurotransmitters metabolism in our experiments. Instead, we observed the lower brain levels of pyridoxamine-related compounds and higher adrenaline levels at the whole-brain scale in SHR/NCrl rats. Interestingly, lower peripheral levels of pyridoxamine have been reported in ADHD patients, and pyridoxamine supplementation has been suggested to normalize ADHD symptoms^58–60^. Pyridoxamine derivatives are co-factors of enzymes crucial to the biosynthesis of multiple neurotransmitters such as GABA, glutamate and monoamines^61^ further supporting that the lower pyrixodamine levels observed in our study could be related to modifications in neurotransmitters contents in this animal model of ADHD.

Concerning adrenaline, higher brain concentrations have already been reported in the medulla oblongata in this animal model, along with greater expression of the phenylethanolamine N-methyltransferase, which is responsible for adrenaline biosynthesis^62–67^. Because the adrenergic neurons of the medulla oblongata are involved in the regulation of blood pressure, these higher levels were associated with the development of hypertension elicited by adult SHR rats. Even though our study was conducted before the appearance of hypertension in this animal model^13^, we cannot exclude that the higher adrenaline levels we observed could be involved in the hypertension development in these rats. However, adrenergic neurons do not only send axonal projections in areas regulating autonomic functions, but also to the locus coeruleus and ventral tegmental area containing noradrenergic and dopaminergic neurons known to be involved in the pathophysiology of ADHD^47,68^. Thus, it cannot be excluded that higher adrenaline levels could be related to modifications in dopamine and noradrenaline contents in specific brain regions in this animal model of ADHD.

### SHR/NCrl rats also exhibited unique peripheral metabolomes

In this study, we were able to differentiate SHR/NCrl rats from controls using discriminant models based on blood and urine samples with accuracies and predictive abilities superior to what was obtained in the brain. Besides, 61% of the metabolites involved in the discriminant model in the brain were also involved in the peripheral models, and almost all these metabolites were significantly different between SHR/NCrl rats and controls in central and peripheral compartments. This good coverage of the brain by the peripheral metabolomes should enable to identify valuable peripheral biomarkers related to the neurobiology underpinning this animal ADHD model. At the scale of metabolomic pathways, the urine compartment shared almost 80% of the differences detected in the blood. The interest of looking at the urine compartment in the context of neuropsychiatric diseases has already been highlighted, as this compartment is not subjected to the homeostatic changes observed in the blood^69^. Since a large number of metabolites are concentrated in urines, this could result in an enhanced sensitivity for the detection of low metabolic variations. In addition, the easy access to urines in humans makes it a compartment of choice for patients in which blood sampling can be more complex to collect. Thus, this further highlight the complementarity between peripheral compartments, and the interest at looking at multiple peripheral matrices to better reflect a cerebral pathophysiological condition.

As in the brain, our blood and urine results revealed significantly modified pathways related to amino acids, energetic, and vitamin metabolisms along with oxidative stress in SHR/NCrl rats vs controls. Clinical studies already explored peripheral matrices such as plasma, serum, saliva, and urine in ADHD subjects. Most of these studies have examined levels of growth factors, cortisol, melatonin, catecholamines, fatty acids, amino acids, oxidative stress, and pyridoxine metabolisms in ADHD patients, but no reliable biomarkers of ADHD have been uncovered to date^7,8,70,71^. However, studies showed results consistent with our findings: (i) impaired responses to oxidative stress have been observed in blood samples of ADHD patients, along with a relationship between low levels of pyridoxine and ADHD;^47,58,71,72^ (ii) despite inconsistent results regarding tryptophan pathway changes in the blood of ADHD children^61,62^, lower serum levels of tryptophan and related metabolites have been reported in adult patients^73^, in line with the association between altered tryptophan and the ADHD-like behaviors exhibited by dogs^60^, and as we observed in SHR/NCrl rats. Taken together, this further support the interest of exploring distinct peripheral matrices and metabolic pathways related to energy metabolism, oxidative stress, vitamins, and amino acids in the search for biomarkers of ADHD.

Questioning the relevance of potential peripheral metabolites of interest in ADHD, animal models represent an interesting opportunity to have access to both central and peripheral compartments. In addition to common differences observed at the scale of metabolic pathways between the brain and periphery of SHR/NCrl rats, we also detected metabolites that were significantly modified in the brain and periphery between SHR/NCrl rats and controls. These metabolites were related to energy metabolism and oxidative stress reinforcing the interest of investigating these metabolic pathways in peripheral compartments in the search of metabolic signature of ADHD. Significant correlations between the brain and urine variations of malonate and glucuronate were also specifically detected in SHR/NCrl rats. Such a relationship is of a great interest as these results emphasize the relevance to look at the whole individual, using cerebral but also peripheral matrices to better understand the biological modifications underlying pathological behaviors.

## Conclusion

Fifteen years ago, Russell and colleagues proposed the so-called “Neuroenergetic hypothesis” of ADHD^52^. This hypothesis suggested alterations in neuronal transmissions due to deficits in energy supply to explain moment-to-moment fluctuations in task performance, which is a common feature of ADHD^52^. Our results are consistent with this hypothesis and give new insights into the molecular mechanisms underlying such energetic dysfunction. Based on the limited access to brain metabolites in humans, the use of animal models to better understand the molecular mechanisms underlying ADHD pathogenesis remains an important avenue of research. The presence of such metabolomic modifications both in the brain and in the peripheral compartments, further support the relevance to look at these common metabolites and related metabolic pathways in the search of potential clinical ADHD biomarkers.

## Supplementary information

Supplementary legends

Table S1

Table S2

Table S3
