# Supplementary material for: SHR/NCrl rats as a model of ADHD can be discriminated from controls based on their brain, blood, or urine metabolomes
Source: Transl Psychiatry. 2021 Apr 22;11:235. doi: 10.1038/s41398-021-01344-4 (PMC8062531; doi:10.1038/s41398-021-01344-4)
Supplement: Supplementary file 1 — Supplementary legends [file 41398_2021_1344_MOESM1_ESM.docx]

**Legends for Supplementary Tables**

**Table S1** List of the discriminant metabolites involved in the PLS-DA model built from brain samples. The variable importance in projection score, p-value corrected for false discovery rate (FDR), and fold change (FC) is noted for each metabolite.

**Table S2** List of the discriminant metabolites involved in the PLS-DA model built from blood samples. The variable importance in projection score, p-value corrected for FDR, and FC is noted for each metabolite.

**Table S3** List of the discriminant metabolites involved in the PLS-DA model built from urine samples. The variable importance in projection score, p-value corrected for FDR, and FC is noted for each metabolite.
