# Supplementary material for: SHR/NCrl rats as a model of ADHD can be discriminated from controls based on their brain, blood, or urine metabolomes
Source: Transl Psychiatry. 2021 Apr 22;11:235. doi: 10.1038/s41398-021-01344-4 (PMC8062531; doi:10.1038/s41398-021-01344-4)
Supplement: Supplementary file 2 — Table S1 [file 41398_2021_1344_MOESM2_ESM.pdf]

Brain VIPs	VIP Score	FDR	FC
2-Deoxygalactopyranose	1.183	-	0.92
2-Ethylmalonate	1.848	<0.0001	5.68
3-Hydroxybutyrate	1.822	<0.0001	4.24
3-Methylhistidine	1.265	0.0496	0.84
4-Guanidinobutanoate	1.427	0.0172	0.83
4-Hydroxy-proline	1.231	-	0.76
4-Trimethylammoniobutanoate	1.579	0.0046	1.39
5-Aminolevulinate	1.231	-	0.76
Adenine	1.262	0.0496	0.85
ADP-glucose	1.313	0.0383	1.15
Anserine	1.146	-	0.89
Glucose	1.169	-	0.90
Glucuronate	1.464	0.0134	1.50
Diaminopimelate	1.330	0.0343	0.86
Epinephrine	1.380	0.0252	1.35
GABA	1.582	0.0046	0.85
Glyceraldehyde-3-Phosphate	1.476	0.0125	0.85
Glyceryl monoacetate	1.431	0.0172	0.66
Glycine	1.118	-	0.86
Guanidoacetate	1.145	-	0.86
Guanosine monophosphate	1.117	-	0.80
Histamine	1.044	-	0.84
Inosinate	1.671	0.0018	0.72
Arginine	1.067	-	0.90
Asparagine	1.789	0.0001	1.11
Aspartate	1.266	0.0496	0.90
Carnitine	1.557	0.0058	1.34
Glutamate	1.193	-	1.08
Lysine	1.603	0.0041	0.67
Phenylalanine	1.154	-	0.93
Proline	1.044	-	1.06
Serine	1.214	-	1.06
Lumichrome	1.270	0.0496	0.87
Maleimide	1.368	0.0256	0.93
Malonate	1.580	0.0046	4.84
Myo-Inositol	1.047	-	0.82
Myristate	1.174	-	0.89
N-Acetyl-acetylneuraminate	1.024	-	0.95
N-Acetyl-L-aspartate	1.616	0.0038	0.95
N-Acetylglutamate	1.073	-	0.95
N-Acetylserine	1.193	-	1.08
N6-Δ2-Isopentenyl-adenine	1.086	-	1.21
Niacinamide	1.538	0.0070	0.94
Nicotinate	1.368	0.0256	0.91
NNN-Trimethyl-lysine	1.637	0.0030	0.72
Pyridoxal-5-phosphate	1.702	0.0011	0.75
Pyridoxamine	1.439	0.0167	0.72

Spermine	1.132	-	0.94
Succinate	1.517	0.0086	1.48
Thiamine monophosphate	1.484	0.0121	0.77
Trans-Aconitate	1.067	-	0.90
Trigonelline	1.379	0.0252	0.83
Ureidopropionate	1.788	0.0001	1.11
