# Supplementary material for: SHR/NCrl rats as a model of ADHD can be discriminated from controls based on their brain, blood, or urine metabolomes
Source: Transl Psychiatry. 2021 Apr 22;11:235. doi: 10.1038/s41398-021-01344-4 (PMC8062531; doi:10.1038/s41398-021-01344-4)
Supplement: Supplementary file 3 — Table S2 [file 41398_2021_1344_MOESM3_ESM.pdf]

Blood VIPs	VIP score	FDR	FC
1-Aminocyclopropanecarboxylate	1.003	-	0.88
3-Dehydroshikimate	1.263	-	1.45
3-Hydroxybutyrate	1.189	-	1.68
3-methyl-2-oxindole	1.843	<0.0001	0.29
3-Methylhistidine	1.887	<0.0001	1.78
4-Hydroxyproline	1.029	-	1.09
5-Aminolevulinate	1.029	0.0302	1.09
5-Aminopentanoate	1.112	0.0094	1.10
Adipate	1.297	0.0003	0.81
Anserine	1.471	0.0414	0.60
Azelate	1.476	-	1.50
Benzyl alcohol	1.724	-	0.18
Beta-Alanine	1.245	0.0178	1.16
Betaine	1.112	<0.0001	1.10
Citrulline	1.064	-	1.20
Creatine	1.377	-	1.26
Creatinine	1.846	0.0321	0.71
Cytosine	1.008	0.0092	0.90
Deoxyuridine	1.013	0.0219	0.65
Diaminopimelate	1.286	-	0.72
Dihydrouracil	1.481	<0.0001	0.56
Formyl-L-Methionyl	1.341	-	1.34
Galactarate	1.141	-	1.55
GABA	1.895	-	0.55
Glucarate	1.141	-	1.55
Gluconate	1.157	0.0185	1.24
Glycine	1.035	-	1.14
Hippurate	1.162	-	1.35
Hydroxyphenyllactate	1.368	0.0185	1.46
Indoleacetaldehyde	1.565	0.0034	1.56
Indoleacetate	1.502	0.0076	2.14
Inosine	1.203	-	1.95
Alanine	1.245	0.0414	1.16
Asparagine	1.352	0.0209	0.79
Glutamine	1.347	0.0214	0.89
Kynurenine	1.876	<0.0001	0.34
Lactate	1.117	-	0.83
Lysine	1.675	0.0006	0.71
Phenylalanine	1.637	0.0012	0.75
Pipecolate	1.725	0.0003	0.61
Threonine	1.463	0.0097	0.88
Tryptophan	1.415	0.0139	0.78
Valine	1.112	-	1.10
Malonate	1.709	0.0004	0.47
Methyl indole-3-acetate	1.376	0.0178	0.72
N-Acetylneuraminate	1.447	0.0112	1.48
N-Acetylserotonin	1.924	<0.0001	0.18

N-Methyltryptamine	1.814	<0.0001	0.22
N6-Δ2-Isopentenyl-adenine	1.418	0.0139	1.52
Nicotinate	1.068	-	1.26
NNN-Trimethyl-lysine	1.877	<0.0001	0.64
Norvaline	1.112	-	1.10
Pantothenate	1.404	0.0151	0.79
Paraxanthine	1.218	0.0483	1.38
Phosphorylcholine	1.298	0.0302	0.60
Pyridoxamine	1.678	0.0006	0.70
Serotonin	1.127	-	1.30
Spermidine	1.437	0.0120	0.68
Sphinganine	1.053	-	2.27
Succinate	1.213	0.0487	0.82
Theobromine	1.182	-	1.37
Theophylline	1.218	0.0483	1.38
Thymine	1.386	0.0174	0.82
Trans-Ferullate	1.025	-	1.36
Trigonelline	1.596	0.0023	0.66
Uracil	1.150	-	0.67
Xanthine	1.197	-	2.10
