# Supplementary material for: SHR/NCrl rats as a model of ADHD can be discriminated from controls based on their brain, blood, or urine metabolomes
Source: Transl Psychiatry. 2021 Apr 22;11:235. doi: 10.1038/s41398-021-01344-4 (PMC8062531; doi:10.1038/s41398-021-01344-4)
Supplement: Supplementary file 4 — Table S3 [file 41398_2021_1344_MOESM4_ESM.pdf]

Urine VIPs	VIP score	FDR	FC
Aminocyclopropanecarboxylate	1.497	<0.0001	0.28
1H-Indole-3-acetamide	1.428	<0.0001	0.22
2-Amino-2-deoxy-gluconate	1.107	0.0033	2.87
2-Aminophenol	1.431	<0.0001	0.24
2-Deoxygalactopyranose	1.124	0.0027	6.95
2-Ethylmalonate	1.127	0.0027	0.50
2.3-Dihydroxybutanedioate	1.215	0.0007	0.41
3-Hydroxybutyrate	1.233	0.0005	0.35
3-Hydroxymethylglutarate	1.337	0.0001	0.41
3-Methyl-2-Oxindole	1.382	<0.0001	0.18
3-Methylhistidine	1.349	<0.0001	2.32
4-Guanidinobutanoate	1.086	0.0043	1.44
4-Hydroxycinnamate	1.274	0.0002	0.32
4-Hydroxyproline	1.041	0.0072	0.52
5-Aminolevulinate	1.295	0.0001	3.75
5-Hydroxy-tryptophan	1.476	<0.0001	22.28
5-Hydroxyindoleacetate	1.367	<0.0001	0.19
5-Hydroxymethyluracil	1.171	0.0015	1.46
5-Methylcytosine	1.051	0.0064	1.62
5-Thymidylate	1.245	0.0004	0.27
5'-Methylthioadenosine	1.029	0.0080	0.57
Acetylcysteine	1.430	<0.0001	0.44
Adipate	1.406	<0.0001	0.32
Aminoadipate	1.165	0.0016	0.74
Benzyl alcohol	1.393	<0.0001	0.15
Beta-Alanine	1.360	<0.0001	0.61
Cadaverine	1.342	<0.0001	0.48
Catechol	1.201	0.0009	0.42
Citramalate	1.185	0.0012	0.55
Creatine	1.324	0.0001	2.91
Creatinine	1.106	0.0033	1.64
Cytidine	1.088	0.0043	3.14
Cytosine	1.324	0.0001	2.32
Glucose	1.265	0.0003	0.22
Glucuronated	1.127	0.0027	2.48
Deoxycytidine	1.134	0.0025	2.22
Deoxyguanosine	1.219	0.0007	0.36
Diethanolamine	1.300	0.0001	2.27
Dihydrouracil	1.368	<0.0001	0.63
Dimethylbenzimidazole	1.134	0.0025	1.54
Divalonate	1.066	0.0054	0.69
DL-O-Phosphoserine	1.190	0.0011	3.94
dUMP	1.117	0.0030	0.35
Gluconate	1.140	0.0023	2.46
Glyceraldehyde-3-Phosphate	1.110	0.0032	1.71
Guanidinosuccinate	1.255	0.0003	4.00
Guanidoacetate	1.487	<0.0001	7.19

Histamine	1.124	0.0027	0.29
Imidazoleacetate	1.303	0.0001	0.61
Indoleacetate	1.204	0.0009	2.48
Kynurenate	1.368	<0.0001	0.44
Asparagine	1.340	<0.0001	0.64
Carnitine	1.296	0.0001	3.12
Cystine	1.358	<0.0001	6.70
Dihydroorotate	1.423	<0.0001	3.83
Glutamate	1.200	0.0010	2.96
Histidine	1.048	0.0066	2.18
Histidinol	1.405	<0.0001	2.77
Lysine	1.307	0.0001	2.07
Malate	1.328	0.0001	0.24
Pipecolate	1.290	0.0002	0.61
Proline	1.193	0.0011	1.80
Serine	1.229	0.0006	0.57
Threonine	1.228	0.0006	2.86
Tyrosine	1.150	0.0020	1.53
Malonate	1.160	0.0018	1.98
Methylglutarate	1.406	<0.0001	0.32
N-Acetyl-acetylneuraminate	1.117	0.0030	1.44
N-Acetyl-glucosamine	1.054	0.0063	0.53
N-Acetyl-methionine	1.010	0.0100	0.65
N-Acetyl-phenylalanine	1.372	<0.0001	0.15
N-Acetylglutamate	1.080	0.0046	0.59
N-Acetylleucine	1.348	<0.0001	0.34
N-Acetylserine	1.157	0.0018	0.65
N-Acetylserotonin	1.423	<0.0001	0.28
N-acetyltryptophan	1.032	0.0078	0.61
N-Formyl-methionine	1.334	0.0001	0.32
N6-Δ2-Isopentenyl-adenine	1.263	0.0003	0.29
Nicotinate	1.302	0.0001	0.31
Phenylacetate	1.194	0.0010	0.55
Pterin	1.380	<0.0001	2.52
Quinate	1.286	0.0002	0.40
Taurine	1.124	0.0027	6.95
Trans-Cyclohexane-diol	1.366	<0.0001	0.23
Trigonelline	1.328	0.0001	0.70
Tryptophanol	1.456	<0.0001	0.16
Uracil	1.080	0.0046	0.75
Ureidopropionate	1.340	<0.0001	0.64
Urocanate	1.039	0.0073	1.55
Xanthurenate	1.464	<0.0001	0.23
